# Stereotactic Radiosurgery for Glioblastoma

**DOI:** 10.7759/cureus.413

**Published:** 2015-12-17

**Authors:** Kristin J Redmond, Minesh Mehta

**Affiliations:** 1 Department of Radiation Oncology and Molecular Radiation Sciences, The Johns Hopkins University School of Medicine, Baltimore, Maryland; 2 Department of Radiation Oncology, University of Maryland

**Keywords:** stereotatic radiosurgery, brain tumor, gamma knife radiosurgery, recurrent glioblastoma, glioblastoma multiforme

## Abstract

Glioblastoma (GBM) is the most common primary malignant brain tumor in adults and one of the most aggressive of all human cancers. GBM tumors are highly infiltrative and relatively resistant to conventional therapies. Aggressive management of GBM using a combination of surgical resection, followed by fractionated radiotherapy and chemotherapy has been shown to improve overall survival; however, GBM tumors recur in the majority of patients and the disease is most often fatal. There is a need to develop new treatment regimens and technological innovations to improve the overall survival of GBM patients. The role of stereotactic radiosurgery (SRS) for the treatment of GBM has been explored and is controversial. SRS utilizes highly precise radiation techniques to allow dose escalation and delivery of ablative radiation doses to the tumor while minimizing dose to the adjacent normal structures. In some studies, SRS with concurrent chemotherapy has shown improved local control with acceptable toxicities in select GBM patients. However, because GBM is a highly infiltrative disease, skeptics argue that local therapies, such as SRS, do not improve overall survival. The purpose of this article is to review the literature regarding SRS in both newly diagnosed and recurrent GBM, to describe SRS techniques, potential eligible SRS candidates, and treatment-related toxicities. In addition, this article will propose promising areas for future research for SRS in the treatment of GBM.

## Introduction and background

Glioblastoma (GBM) is the most common primary malignant brain tumor and is one of the most aggressive of all human cancers. The National Cancer Institute estimates that there will be 23,380 newly diagnosed brain or other nervous system tumors in 2014, with an estimated 14,320 deaths [1]. GBM accounts for approximately 15% of all brain tumors and primarily occurs in adults between the ages of 45 and 70. The symptoms of GBM vary depending on the location of the tumor but may include persistent headaches, seizures, vision changes, nausea, vomiting, loss of appetite, difficulty with speech, changes in mood and behavior, mental capacity and concentration, cranial nerve deficits, and motor or sensory abnormalities. The putative cell of origin of this rapidly proliferating tumor with a high growth fraction is the astrocyte, and the World Health Organization (WHO) classifies this as a Grade IV astrocytoma. These tumors are generally highly infiltrative and relatively resistant to conventional therapies, such as surgery, radiation therapy, and chemotherapy. The disease is generally associated with rapid progression and fatal outcomes, typically resulting in death in the first 15 months or less after diagnosis, with very small numbers of long-term survivors. 

The standard of care following maximal safe resection is fractionated radiation therapy to 60 Gray (Gy) in 30 fractions, plus temozolomide chemotherapy (concomitant with radiation therapy, and adjuvantly for six monthly cycles) based on a randomized controlled trial, which demonstrated a significant improvement in overall survival compared to patients receiving radiation therapy alone [2-3]. In spite of aggressive management, this tumor recurs in the majority of patients and the disease is almost universally fatal. Given the poor prognosis, developing innovative new methods for managing this disease are essential. 

Whereas conventionally fractionated radiation therapy allows safe treatment of large volumes by taking advantage of the differential rates of repair between normal tissue and tumor cells, stereotactic radiosurgery (SRS) utilizes highly precise radiation techniques to allow dose escalation and delivery of ablative radiation doses to the tumor while minimizing dose to the adjacent normal structures. Recent technological advances allow submillimeter precision and the possibility of fractionation. 

The role of SRS in the management of glioblastoma is controversial [4]. Radiation therapy is the single most effective adjuvant therapy following surgical resection for GBM. Studies have shown that GBM recurrences predominantly occur within a 2 cm border of the original tumor following resection and radiation therapy [5]. Given that GBM recurrences are predominantly local, proponents of radiosurgery note that it allows dose escalation while limiting exposure to adjacent critical structures. Skeptics report that GBM is a highly infiltrative disease that extends beyond the radiographically apparent margins, making utilization of a highly conformal technique counterintuitive. 

The purpose of this manuscript is to review the literature regarding SRS in both newly diagnosed and recurrent GBM and to describe the technique as well as potential candidates for it. In addition, we will discuss toxicities and propose promising areas for future research.   

## Review

### SRS in newly diagnosed GBM

The only Level I data evaluating the efficacy of SRS in patients with newly diagnosed GBM comes from RTOG 9305, which randomized 203 patients with supratentorial GBM measuring less than 4 cm to external beam radiation therapy (60 Gy), plus bis-chloroethylnitrosourea (BCNU) chemotherapy with or without an upfront SRS boost [6]. The SRS radiation doses were dependent on the tumor size but ranged from 15 to 24 Gy delivered in a single fraction. There was no difference in median overall survival, two and three-year overall survival rates, or in the pattern of failure between the two treatment groups. In addition, quality of life and cognitive outcomes were comparable between the two groups. 

Compellingly negative as these results are, they are less directly applicable to the current standard of care management, which involves concurrent and adjuvant temozolomide, a regimen more likely to control infiltrative microscopic disease beyond the SRS boost region [2-3]. Local failure in GBM is likely a consequence of at least two separate phenomena: one representing inadequate dose (which can be rectified with an SRS boost), and the other representing geographic failure due to microscopic extension beyond the SRS margin (which currently can only be addressed with temozolomide). Because SRS can influence only one of these failure mechanisms, a contemporary trial combining temozolomide with an SRS boost is called for. In fact, in one report from UCSF where GBM patients undergoing SRS were evaluated with MR spectroscopy (MRS), patients with significant abnormal magnetic resonance spectroscopy (MRS) outside the SRS dose region relapsed rapidly and appear not to benefit, implying the need for another therapeutic approach to control infiltrative microscopic disease at some distance from the SRS dose margin [7]. Some critics have suggested that in RTOG 9305 SRS was delivered *prior* to conventional radiation therapy, and that perhaps the alternative strategy to consider is to complete conventional radiotherapy and temozolomide and use SRS boost as a randomized comparator only in those patients whose disease has not progressed, thereby allowing the selection of a cohort of patients most likely to benefit from SRS [8]. 

Another multi-institutional study, RTOG 0023, a Phase II study, enrolled 76 patients with newly diagnosed GBM with < 6 cm of residual contrast-enhancing tumor and provided intriguing insights for patient selection [8]. In this trial, patients were treated with 50 Gy conventional radiation therapy with four fractionated stereotactic radiosurgery boost fractions of either 5 or 7 Gy administered once weekly during the final four weeks of therapy. This study also used BCNU, rather than temozolomide. While the regimen was safe, there was no survival benefit compared to the historical RTOG database for the entire cohort; however, the patient subset undergoing complete or near-complete resection, in fact, did appear to have improved survival with this boost approach, suggesting that perhaps minimal to no residual disease might represent an important selection parameter. 

In contrast, a number of retrospective reports as well as single institution prospective studies evaluating SRS in combination with EBRT, with or without chemotherapy, including temozolomide, have suggested a potential local control or overall survival benefit with variable, but generally acceptable, toxicity [9-19]. See Table 1 for a summary of published results for SRS treatment of newly diagnosed glioblastoma. 

**Table 1:** Summary of Published Studies of SRS for the Treatment of Newly Diagnosed Glioblastoma Abbreviations: AA: anaplastic astrocytoma; DSS: disease-specific survival; GBM: glioblastoma multiforme; KPS: Karnofsky Performance Score; NR: data not reported; OS: overall survival; PFS: progression-free survival; RT: external beam radiation therapy; SRS: stereotactic radiosurgery

Author	N	Treatment Schema	Survival Rate	Median OS (months)
Sarkaria [9]	115	54-60 Gy RT + 6-20 Gy SRS	2-yr OS: 45% 2-yr OS for KPS ≥ 70: 51% 2-yr OS for KPS < 70: 0%	NR
Gannett [10]	30	44-62 Gy RT + 0.5-18 Gy SRS	1-yr DSS: 57% 2-yr DSS: 25%	13.9
Masciopinto [11]	31	RT + 15-35 Gy SRS	1-yr OS: 37%	9.5
Mehta [12]	31	54 Gy RT + 15-30 Gy SRS	1-yr OS: 38% 2-yr OS: 28%	42 weeks
Nwokedi [13]	33 RT alone; 31 RT + SRS	28-80 (median 59.7) Gy RT + 10-28 (median 7.1 Gy) SRS	For all patients: 1-yr OS: 67% 2-yr OS: 40% 3 yr OS: 26%	RT alone: 13 RT + SRS: 25
Balducci [14]	41 (36 GBM, 5 AA)	59.4 Gy or 50.4 Gy RT + 10 or 19 Gy SRS (total dose of 69.4 Gy) + temozolomide	2-yr OS: 63%	All pts: 30 GBM: 28
Cardinale [15]	9 GBM 3 AA	44 Gy RT + 36 Gy SRS	NR	GBM: 16 AA: 33
Shrieve [16]	78	RT + SRS	1-yr OS: 88.5% 2-yr OS: 35.9%	19.9
Floyd [17]	20	40 Gy RT + 24 Gy SRS, temozolomide	NR	13
Landy [18]	23	Estramustine + SRS	2-yr OS: 38%	16
Omuro [19]	40	6 x 6 Gy or 6 x 4 Gy SRS + temozolomide + bevacizumab	1-yr OS: 93%	19

A more limited number of small studies have suggested optimistic outcomes following SRS alone in patients with newly diagnosed GBM [20-22]. In aggregate, these data suggest a potential benefit (which obviously needs to be rigorously tested) of SRS with concurrent chemotherapy, preferably temozolomide, in newly diagnosed GBM with favorable prognostic factors, including an extensive resection with limited residual tumor volume, young age, and high Karnofsky performance status (KPS) [23]. Nonetheless, these studies are weakened by their small size and concerns, such as selection bias; therefore, in the absence of additional higher-level data, at present SRS alone or as a boost following external beam radiation therapy is not considered a standard therapy in patients with newly diagnosed GBM. 

One particular SRS approach, popularized under the term “leading-edge radiosurgery” as well as a variation of it, referred to as “border-zone radiosurgery” deserves specific mention. In these approaches, rather than defining the SRS target as a geometric contour of the enhancing abnormality or surgical bed only, an “edge” or “zone” predictive of the highest likelihood of harboring microscopic residual disease predictive of the likelihood of relapse is targeted, using advanced magnetic resonance imaging parameters [24]. Duma, et al. presented a 15-year follow-up in 109 newly diagnosed GBM patients, the majority of whom received upfront involved field radiotherapy and temozolomide, and “leading edge” SRS to a mean volume of 33.5 cc (2.5 - 220 cc range), with a median dose of 8 Gy at the 50% isodose line [Duma, C: Front "leading edge" Gamma Knife radiosurgery for newly diagnosed GBM: a 15-year follow-up. Presented at the 17th International Leksell Gamma Knife Society Meeting, 2014, unpublished data]. The second, third, and fifth-year survival rates were 43, 19, and 12%, respectively. The majority (97/109) of the patients were in the RTOG RPA Class IV, and an indirect comparison with separate radiotherapy alone (n = 150) and radiotherapy and temozolomide (n = 152) revealed a median survival of 13, 16, and 23 months, the latter representing the “leading edge” group.

With advances in technology, the concept of single fraction SRS has started to emerge in certain clinical situations into a hypofractionated approach, combining the principles of stereotaxy and fractionation. This approach, essentially a variant of SRS, has also been explored in the upfront management of GBM with several institutional reports and studies. One intriguing study in this context, by Omuro, et al., treated 40 newly diagnosed GBM patients on an in-house clinical trial with hypofractionated stereotactic radiotherapy (6 Gy x 6 to the contrast-enhancing region and 4 Gy x 6 to the FLAIR abnormality, using a simultaneous integrated boost approach) in combination with temozolomide and bevacizumab, with the latter being used primarily to control peritumoral and radiation-induced edema and vascular changes [19]. The one-year overall survival was 93% and the median survival was 19 months. Similar to the prior RTOG trial, patients undergoing gross total resection fared better with a median survival of 26 months, compared to 16 months for patients with lesser resections. More intriguingly, the median overall survival for unmethylated patients was 22 months, compared to 18 months for methylated tumors. Most conventional non-SRS GBM studies combining temozolomide and radiotherapy describe median survival values of approximately 14 months for the unmethylated subgroup, suggesting that the therapeutic value of hypofractionation might be greatest in the MGMT unmethylated tumors, which are more likely to be mesenchymal and hypoxic [19].

Therefore, from the early era of RTOG 9305, where SRS was used without temozolomide, to the modern era of combination chemotherapy, and the recognition of molecular subtypes that could identify subgroups that could benefit from SRS, a careful and scientifically rigorous reevaluation of the role of this modality is warranted.

### SRS in recurrent GBM

An alternative application of SRS is in patients with recurrent GBM that have progressed following the standard of care fractionated radiation therapy, plus temozolomide. A number of small prospective and retrospective series suggest that SRS may prolong survival in this setting, either alone or in combination with chemotherapy (Table 2) [25-35].  

**Table 2:** Summary of Published Studies of SRS for Recurrent GBM Abbreviations: ANA: anaplastic astrocytoma; ANMO: anaplastic mixed oligoastrocytoma; fx: fraction; GBM: glioblastoma multiforme; Gy: Grey; RT: external beam radiation therapy; SRS: stereotactic radiosurgery; WHO: World Health Organization

Author	N	Treatment Schema	Median Time to 1^st^ Recurrence (Range) Months	OS Rate After SRS Salvage	Median OS (Range) Months
Shrieve [25]	86 tx with SRS alone; 32 tx with brachytherapy alone	13 Gy (median) SRS	NR	1-yr (SRS pts): 45% 2-yr (SRS pts) : 19%	10.2 for SRS pts
Vordermark [26]	19	20-30 Gy SRS	19 (3-116)	1-yr: 26% 2-yr: 16%	9.3 (1.9-77.6+)
Lederman [27]	9 SRS alone; 14 SRS + Taxol	SRS alone: Mean dose 19.2 Gy in 1 fraction SRS + Taxol: Mean dose of 24 Gy in 4 fractions	11	1-yr SRS alone: 11% 1-yr SRS + Taxol: 50%	SRS alone: 6.3 SRS + Taxol: 14.2
Combs [28]	32	10-20 Gy (median 15 Gy)	10 (1-77)	6 months: 72% 1-yr: 38%	10
Fogh [29]	147	28-80 Gy (median dose 35 Gy in 3.5 Gy fractions)	8 (4-205)	NR	11
Maranzano [30]	22	17 Gy (median) SRS or 30 Gy (median) fractionated SRS	9		11
Greenspoon [31]	31	25 – 30 Gy + temozolomide	NR	NR	9
Hudes[32]	20	24 Gy/3 fx 30 Gy/3 fx 35 Gy/3.5 fx	3.1 (0.7-45.5)	1-yr OS: 20%	20
Lederman [33]	88	4 weekly irradiation (median 6 Gy) after Paclitaxel	6.5	1-yr: 17% 2-yr: 3.4%	7
Cuneo [34]	WHO Grade 3: 16 WHO Grade 4: 33	12.5-25 (median 15) Gy SRS 12.5 – 25 Gy SRS + bevacizumab	All pts: 20	WHO Grade 3, 1-yr: 22% WHO Grade 4, 1-yr: 50%	WHO Grade 3 glioma: 3.9 WHO Grade 4 glioma: 11.2
Minniti [35]	54	30 Gy/6 fx SRS + temozolomide	Median time between primary RT and reirradiation: 15.5	1-yr: 53% 2-yr: 10%	12.4

In these studies, the median time from the initial course of radiation therapy to radiosurgery varied (range: 3-10 months) and overall survival from the time of reirradiation ranged from 7-16 months, which generally compares favorably to the anticipated survival in patients with recurrent GBM. In most situations, SRS is performed once in a patient’s disease course; however, there is a case report of multiple rounds of SRS to different regions of recurrence in a patient with a prolonged survival [36].

Recently, Larson, et al. reviewed the literature for recent studies describing the use of Gamma Knife radiosurgery for recurrent GBM and identified nine reports between 2005-2013 (Table 3) [37]. The median overall survival ranged from 9^_^17.9 months from salvage SRS, and the median progression-free survival ranged from 4.6^_^14.9 months (Table 3) [38-46]. 

**Table 3:** Summary of Outcomes Following Salvage Gamma Knife SRS for Recurrent GBM Abbreviations: BZ: Bevacizumab; EF: extended field; GK: Gamma Knife; GTR: gross tumor resection; NR: data not reported or not segregated for GBM patients

Author	N	Median SRS Dose (Range) Gy	Time to 1^st^ Recurrence (Months)	Median Survival From Diagnosis (Months)	Median Survival After Treatment Months)	Local Tumor Control
Skeie [38]	32 GK; 26 reoperation; 19 both procedures	12.2 (8-20)		51 pts that received GK: 19 26 pts with reoperation only: 16	51 pts that received GK: 12 26 pts with reoperation only: 6	
Park [39]	11 GK + BZ; 44 GK alone	16 (13-18) 15 (10 -20)	NR	33.2 26.7	17.9 12.2	NR NR
Koga [40]	9 conventional GK; 9 EF GK	20 20	14.5 (median) 12 (median)	24 21	10.5 9	47% 93%
Elliott [41]	16	16	7.9 (median)	26.1	13	NR
Pouratian [42]	26	26	NR	17.4	9.4	NR
Kida [43]	54	54	NR	27.0	14.0	NR
Kong [44]	65	65	4.3	23.0	13	NR
Kohshi [45]	11	11	NR	21.0	11	NR
Hsieh [46]	26	26	NR	16.7	10	NR

A challenge in interpreting these and other results is that many studies do not report details regarding the most relevant factors. For example, factors, such as initial radiation dose, the extent of initial and second surgical resections, tumor volume at the time of SRS, timing and use of chemotherapy, and the time between initial radiation therapy and retreatment have clear implications on patient outcomes but are variably reported [47]. In addition, many studies do not report on re-operation following SRS and whether the final pathology from that intervention revealed active tumor versus radiation necrosis. Finally, selection bias could also contribute to better than expected results in these reports. Nevertheless, in spite of all of these limitations, it appears that a small proportion of patients do live longer than expected and careful evaluation of the role of SRS in the recurrent setting is warranted. In this context, the “border zone” recurrent GBM trial recently initiated by the Gamma Knife Consortium represents a well-coordinated multi-institutional effort designed to prospectively and rigorously study this therapy [48]. Patients will be treated with "border-zone" SRS where magnetic resonance spectroscopy (MRS) will be used to guide the treatment volume in a single arm Phase II study. Patients will be treated with bevacizumab prior to SRS and then every 14 days until the time of tumor progression. The primary endpoint is a two-year overall survival.  

Similar to the experience with newly diagnosed GBM, several small prospective and retrospective studies have evaluated the addition of bevacizumab to SRS in recurrent GBM. The results have been favorable with overall survival ranging from 12-15 months, one-year survival as high as 50%, and lower rates of adverse radiation effects (range: 0-10%) [34, 39, 49].

### Toxicity of SRS

A concern with SRS for GBM in both the upfront and recurrent setting is the high rate of toxicity. Several studies have reported serious neurologic deficits, including hemiparesis, severe headache, somnolence, and vision loss following SRS [9, 16, 29, 34-35, 39, 49-53]. Rates of reoperation are high, ranging from 50-57%, and reveal high rates of radiation necrosis (range: 10-38%) [9, 16, 23, 54]. In addition, prolonged requirements for corticosteroids have been reported in a large percentage of patients [9, 55]. See Table 4 for a summary of toxicities following SRS treatment of GBM. 

**Table 4:** Summary of Toxicities for SRS Treatment of GBM Abbreviations: AA: anaplastic astrocytoma; CNS: central nervous system; fx: fraction; Gy: Grey; SRS: stereotactic radiosurgery

Author	N	Dose	Toxicity
Sarkaria [9]	115	54 – 60 Gy RT + 10 – 20 Gy SRS	17 patients with radiation necrosis, 1 patient with hemiparesis. 47% required prolonged steroid use. One patient with double vision and hydrocephalus requiring ventricular shunt.
Schrieve [16]	78		50% had reoperation for symptomatic necrosis or recurrent tumor. Rate of reoperation at 24 months after SRS was 54.8%.
Fogh [29]	147	Median 35 Gy/3.5 Gy fx	One late Grade 3 CNS toxicity 4 months after hypofractionated SRS.
Cuneo [34]	21 SRS 42 SRS + bevacizumab	12.5-25 Gy 12.5 – 25 Gy + bevacizumab	14% Grade 3, 5% Grade 4, 19% radionecrosis, 29% worsening of neurologic symptoms, 19% increase seizures 10% Grade 3, 5% radionecrosis, 24% worsening of neurologic symptoms, 21% increase seizures
Minniti [35]	54	30 Gy/5 fx + temozolomide	7% Grade 3 neurologic deterioration with radiation-induced necrosis; 7 patients with Grade 3 lymphopenia, 3 patients with Grade 4 lymphocytopenia, 2 patients with Grade 3 thrombocytopenia,
Park [39]	11	13-18 Gy + bevacizumab	One Grade 3 toxicity and 1 major adverse radiation effect.
Gutin [49]	25 (20 GBM and 5 AA)	30 Gy/5 fx + bevacizumab	8% Grade 3 leukopenia, 8% Grade 3 neutropenia, 28% Grade 3 lymphopenia, 8% Grade 3 thrombocytopenia, 12% Grade 3 anemia, 4% Grade 3 fatigue, 4% Grade 3 hypertension, 4% Grade 3 CNS hemorrhage, 8% Grade 4 lymphopenia, 4% Grade 4 thrombocytopenia, 4% Grade 4 bowel perforation, 4% Grade 4 wound healing complication, 4% Grade 4 gastrointestinal bleeding
Lederman [50]	14	4.5 – 9 Gy x 4 fx (median 6 Gy x 4) + Taxol	4 patients had re-operation, 3 of 4 had radionecrosis only, 1 of 4 had radionecrosis and tumor detected
Niyazi [51]	20 SRS alone 10 SRS + bevacizumab	36 Gy/18 fx +/- bevacizumab	1 Grade 2 fatigue, 1 Grade 2 hypertension, 1 Grade 3 deep vein thrombosis, 1 Grade 4 wound healing complication
Ogura [52]	30	22.5 – 35 Gy/5 fx	2 patients with Grade 3 radionecrosis
Cabrera [53]	15	18 or 24 Gy/1 fx or 25/5 fx + bevacizumab	1 Grade 3 severe headache, 2 Grade 2 CNS toxicities. No Grade 4 or 5 events.

### SRS, plus bevacizumab, to modulate toxicity

To minimize some of these toxicities, a number of studies, as mentioned above, have combined SRS for GBM with bevacizumab. Bevacizumab is a monoclonal antibody that inhibits vascular endothelial growth factor-A, thereby impeding angiogenesis. While a large randomized prospective study did not find a survival benefit with the addition of bevacizumab to the standard of care therapy in patients with newly diagnosed GBM, it is hypothesized that the drug’s impact on vascular permeability may reduce the risk of SRS-associated edema and necrosis [56]. In fact, a number of small studies have demonstrated that bevacizumab can, in fact, be utilized to treat radiation necrosis, and as mentioned above, when bevacizumab is added to SRS in recurrent GBM, the rates of adverse radiation effects are lower (range: 0-10%) [34, 39, 49, 55]. Similar results were reported in patients receiving bevacizumab with re-irradiation using conventional fractionation [51]. In the newly diagnosed setting, in the above-described Memorial Sloan Kettering Cancer Center study utilizing bevacizumab for newly diagnosed GBM patients treated with hypofractionated stereotactic radiotherapy, there was no reported pseudoprogression with an encouraging quality of life and neuropsychological evaluations. Nonetheless, bevacizumab is associated with its own substantial toxicities, including intratumoral hemorrhage and bowel dehiscence [49]. While the results are favorable, further prospective investigations are necessary before the treatment paradigm can be broadly applied in clinical practice. 

### Patient selection

The role of SRS in patients with glioblastoma remains controversial. At present, there are minimal data to support its unselected use in the newly diagnosed setting, although further investigation of new paradigms, such as the use of concurrent bevacizumab with SRS and temozolomide, remains warranted. Similarly, there are no consensus guidelines to direct practice in the recurrent setting. However, most studies have enrolled patients with limited disease measuring < 4-5 cm in maximal diameter and a minimum of 5-10 millimeters from the tumor to dose-limiting critical structures, such as the optic apparatus. Care should be utilized in patients with gross disease involving the brainstem and patients with subependymal spread of their disease, who are less likely to benefit from the procedure. The ideal candidates for SRS in recurrent GBM would have a favorable performance status, good response to initial chemoradiation therapy, a relatively prolonged interval to recurrence, and limited volume, circumscribed recurrence. At least one report suggests that MRS could be utilized to select appropriate candidates, and PET imaging with novel tracers could also have a role in this situation. It should be noted that the radiographic appearance of pseudoprogression and necrosis mirrors tumor progression, which needs to be ruled out prior to proceeding with SRS. 

### SRS technique

The frame-based approach is the most common technique utilized for single fraction radiosurgery and affords unparalleled accuracy. Fractionated delivery is often performed with the patient immobilized supine in an aquaplast mask. A contrast-enhanced high-resolution thin slice (e.g. 1 mm) MRI should be considered the requisite baseline image for contouring purposes and is often co-registered to a simulation computerized tomography (CT), which, if performed, should similarly also be a thin-slice dataset. Many institutions define the gross tumor volume (GTV) as the region of contrast enhancement on T1 post-gadolinium MRI, with minimal or no margin. In other words, clinical target volume (CTV) = GTV; for framed systems, the planning target volume (PTV) = CTV, but a set-up margin for PTV expansion is often used for mask-based systems. However, retrospective data, at least with the fractionated approach, suggest improved local control when FLAIR abnormalities are included in the target volume or when larger margins of 0.5 to 1 cm are utilized [57-58]. These studies suggest that the extended treatment volumes have acceptable toxicity and a benefit in terms of both local tumor control and regional tumor progression. It remains unclear whether this will translate into a survival benefit, but the concept, potentially with concurrent bevacizumab, is worthy of further investigation.    

There is wide variability in dose and fractionation schedules between institutions and studies. In patients with disease amenable to treatment with a single fraction, the doses outlined in RTOG 9005 are reasonable. According to this study, the maximum tolerated single fraction dose for patients with tumors ≤ 20 mm is 24 Gy, 21-30 mm is 18 Gy, and 31-40 mm is 15 Gy [59]. In patients with larger tumors or where there is concern that edema might lead to worsening neurologic deficits, fractionation is advisable. Reasonable fractionation schemes include 25-35 Gy in five fractions [31, 53]. A dose response has been noted, with improved local control with higher radiation doses, but the optimal schedule remains to be determined [32]. 

The dose-limiting structures are generally the optic apparatus and brainstem. Although many institutions are more conservative in their dosing, for patients without prior radiation receiving a single fraction treatment, the maximum point dose to the optic apparatus according to AAPM Task Group 101 (TG-101) is 10 Gy and to the brainstem is 15 Gy [60]. In patients receiving five fractions, TG-101 allows a maximum point dose to the optic apparatus of 25 Gy and 30-31 Gy to the brainstem [60]. In the retreatment setting, the constraints for these structures should be modified to account for the previous dose. 

### Future directions

There are a variety of critical questions that remain regarding the role of SRS in GBM. As discussed above, the optimal fractionation schedule and target volumes remain uncertain. Innovative imaging technologies to more definitively delineate the region of the active tumor may allow improved outcomes while potentially reducing toxicity. For example, a recent Phase II study utilizing MRS for SRS target definition revealed a significantly improved median overall survival compared to a historical control [60]. Techniques that actively identify infiltrative tumor margins, as opposed to iso-volumetric expansions, might prove to be useful as suggested by the “leading-edge” and “border-zone” concepts.

Furthermore, the potential role of bevacizumab or other systemic agents to mitigate the toxicities of SRS will be an important avenue of future study. Similarly, case reports have suggested an abscopal effect in patients receiving immune checkpoint inhibitors with radiation therapy, and a case report from a patient with brain metastases from melanoma suggested complete resolution on follow-up imaging [62-63]. While prospective data evaluating this concept in patients with GBM do not exist currently, these combination therapies may provide an intriguing approach to treating this tumor.

## Conclusions

GBM is one of the most aggressive human malignancies, which remains almost universally fatal in spite of aggressive multimodality therapy including surgery, radiation therapy, and chemotherapy. The predominant pattern of recurrence is local, and SRS represents a technique to precisely deliver high doses of radiation while limiting exposure to the adjacent critical structures, such as the brainstem and optic chiasm. At present, the technique is most appropriate in patients with focally recurrent GBM. Preliminary studies utilizing bevacizumab in combination with SRS suggest that it may help to mitigate SRS toxicity in this setting. Future investigations will be critical in further evaluating this possibility and determining the optimal dose and fractionation schedules.
